# The Effect of Astaxanthin-Rich Microalgae “Haematococcus pluvialis” and Wholemeal Flours Incorporation in Improving the Physical and Functional Properties of Cookies

**DOI:** 10.3390/foods6080057

**Published:** 2017-07-26

**Authors:** A. K. M. Mofasser Hossain, Margaret A. Brennan, Susan L. Mason, Xinbo Guo, Xin An Zeng, Charles S. Brennan

**Affiliations:** 1 Centre for Food Research and Innovation, Department of Wine, Food and Molecular Biosciences, Lincoln University, Lincoln 7647, New Zealand; AKMMofasser.Hossain@lincolnuni.ac.nz (A.K.M.M.H.); Margaret.Brennan@Lincoln.ac.nz (M.A.B.); Sue.mason@lincoln.ac.nz (S.L.M.); 2 Riddet Institute, Palmerston North 4442, New Zealand; 3 School of Food Science and Engineering, South China University of Technology, Guangzhou 510640, China; xbg720@gmail.com (X.G.); xazeng@scut.edu.cn (X.A.Z.)

**Keywords:** microalgae, *Hematococcus pluvialis*, astaxanthin, bakery products, glycaemic response, antioxidant

## Abstract

Marine-based food supplements can improve human nutrition. In an effort to modulate glycaemic response and enhance nutritional aspects, marine-derived algal food rich in astaxanthin was used in the formulation of a model food (wholemeal cookie). Astaxanthin substitution of cookies made from three flours (wheat, barley and oat) demonstrated a significant reduction in the rate of glucose released during in vitro digestion together with an increase in the total phenolic content (TPC) and antioxidant capacity of the food. The significantly (*p* < 0.005) lower free glucose release was observed from cookies with 15% astaxanthin, followed by 10% and then 5% astaxanthin in comparison with control cookies of each flour. Total phenolic content, DPPH radical scavenging and Oxygen Radical Absorbance Capacity (ORAC) value also notably increased with increase in astaxanthin content. The results evidence the potential use of microalgae to enhance the bioactive compounds and lower the glycaemic response of wholemeal flour cookie.

## 1. Introduction

Whole-grains such as wheat, barley and oat make a substantial contribution to our diet. They contain a significant amount of bioactive compounds such as fibre, minerals, vitamins and phytochemicals [1,2] and as such mayplay a major role in enhancing human health by reducing the risk of diabetes [3,4] and cancer [5], while also regulating serum cholesterol [6] and stimulating beneficial gut microbiota [7]. In recent years there has been an increased interest in the utilisation of whole-grain food materials as well as fibre rich ingredients, in cereal products, including bread [8], extruded snack products [9,10], and pasta [11,12]. These pieces of research have investigated the impact of wholegrains and fibre on both the physicochemical characteristics of cereal food products as well as their nutritional quality. A recent review on this subject illustrated that the incorporation of fibre rich ingredients into cereal products often results in negative consumer acceptability [13]. There therefore remains a challenge to both utilise wholegrain cereal products as well as functional food ingredients such a fibre rich materials, into mainstay food products.

Recent research into functional food ingredients has shown an interest in the development of foods containing seaweed or algal materials [14,15]. These materials have been part of the human diet since 600 BC [16] and they have a role of diet in sustaining human due to their diverse range of nutrients and bioactive compounds; such as polysaccharides, proteins, polyunsaturated fatty acids, minerals and significant amounts of antioxidants [17,18]. One such material is *Haematococcus pluvialis,* a single-cell microalgal strain, which is rich source of astaxanthin (10,000–40,000 mg/kg) and associated bioactive ingredients including dietary fibre [19]. Several cell culture and animal studies have reported that astaxanthin has potent antioxidant activity 10 times higher than other carotenoids such as β-carotene, lutein, and zeaxanthin, and 500 times higher than vitamin E [20,21,22]. Carotenoids play a role in preventing or delaying degenerative diseases such as cancer and atherosclerosis diseases [23,24,25], and may be useful in the development of functional foods [15].

There is a paucity of information regarding combining the nutritional compounds of marine-based material and whole-grains. Therefore, the present study is the first to show the glycaemic glucose equivalents (GGE) as a predictor of glycaemic response, antioxidant capacities and physical properties of cereal and *Hematococcus pluvialis* in a model food.

## 2. Materials and Methods

### 2.1. Sample Collection and Preparation

Driedmicroalgae *Hematococcus pluvialis* was provided by Supreme Biotechnologies Ltd. (Nelson, New Zealand) and ground using a grinder (AutoGrinder, M-EM0415, Sunbeam Corp Ltd., Auckland, New Zealand). The ground material was sieved through a 0.5 mm screen to obtain flour. Wholemeal wheat (Champion Flour, Auckland, New Zealand), barley (Ceres Organics, Auckland, New Zealand) and oat flours (Ceres Organics, Auckland, New Zealand) were purchased locally.

### 2.2. Cookie Preparation

Cookies were prepared following the standard American Association of Cereal Chemistry (AACC) method 10–50D [26] with slight modification. Table 1 illustrates the dry ingredients used (sugar, salt and sodium bicarbonate). All dry ingredients (except flour) were mixed in an electric mixer (Breville, Melbourne, Australia) with vegetable shortening (Kremelta, Peerless foods, Braybook, Australia) for 3 min on speed 1. Dextrose solution (8.9 g dextrose anhydrous in 150 mL water) and distilled water were added to the mixer and mixed for a further 1 min on speed 2 with scraping down every 30 s. The flour was added and mixed for 2 min with scraping down every 30 s. The experimental samples were prepared by replacing the wholemeal flour with astaxanthin powder 5%, 10% and 15%. The cookie dough was rolled to a 6 mm thickness using measuring roller and cut with a 57 mm diameter cookie cutter. The cookies were placed on metal trays and baked in a preheated electric oven (BAKBAR turbofan convection oven, E3111, Moffat Pty Ltd., Rolleston, New Zealand) for 8 min at 180 °C. The cookies were cooled at room temperature, placed in air-tight plastic bags and stored at room temperature for 24 h prior to laboratory analysis.

### 2.3. Physical Characteristics

Cookie diameter (mm) and thickness (mm) were measured using calipers (INSIZE digital caliper, series 1112, INSIZE Inc., Loganville, GA, USA). The colour of the cookie samples were measured in terms of Comission Internationale de l’Eclairage (CIE) L*, a* and b* systems by using a colorimeter (Konica Minolta, Chroma Meters CR-210, Tokyo, Japan). The colour differences of the cookies were calculated by the following equation.

$$\Delta E=\sqrt{{\left(\Delta L^{*}\right)}^{2}+{\left(\Delta a^{*}\right)}^{2}+{\left(\Delta b^{*}\right)}^{2}}$$

### 2.4. Texture

The hardness of the cookies (fracture force) was measured by using a texture analyser (TA.XT plus Texture Analyser, Stable Micro Systems, Godalming, UK) with a 3-point bend rig. The analyser was set at a load cell 50 kg; pre-test speed 2 mm/s; test speed 5 mm/s; post-test speed 10 mm/s; return to start mode. The whole cookies were placed on the support ring and the probe moved downward until the samples were broken. The peak force (kg) was recorded as hardness. Measurements were made in triplicate.

### 2.5. Moisture

Moisture content of the cookie samples were measured after drying cookie ground samples (2 g) overnight in an oven at 105 °C.

### 2.6. Determination of Total Phenolic Content

The content of total phenolics of samples was measured by Folin-Ciocalteu reagent (mixture of phosphotungstic and phosphomolybdic acid; that is reduced by phenolics forming a blue complex) using the method described by Floegel et al., 2011 [27] with some modifications. The ground samples (1 g) were dispersed in 20 mL of 70% methanol (by placing on a stirrer overnight). The sample mixture was centrifuged at 700 g Relative centrifugal force (RCF) for 10 min and the supernatant collected to determine the total phenolics. Crude extracts (0.5 mL) were mixed thoroughly with freshly prepared 0.2 N Folin-Ciocalteu’s reagent (2.5 mL), followed by 2.0 mL of 7.5% sodium carbonate (Na₂CO₃) and incubated in the dark for 2 hours. The absorbance reaction mixture was measured at 760 nm. Gallic acid (gallic acid, 97%, CAS: 149-91-7, Sigma-Aldrich, St. Louise, MO, USA) was used as a standard and results were expressed as mg gallic acid equivalent (GAE) per g sample.

### 2.7. Antioxidant Properties

The antioxidant capacity of the samples was measured by the DPPH (2,2-diphenyl-1-picrylhydrazyl) assay as described by Floegel et al., 2011 [27] with some modifications. Briefly, 0.5 mL of crude extract was mixed with freshly prepared 1 mL of 0.1 mM methanolic DPPH (CAS: 1898-66-4, Sigma-Aldrich, St. Louise, MO, USA) solution and incubated in the dark at room temperature for 30 min. The reaction mixture absorbance was measured at 517 nm. In order to calculate the DPPH radical scavenging capacity, trolox (CAS: 53188-07-1, ACROS Organics™, Morris, NJ, USA) was used as a standard and result were expressed as µmol trolox equivalent (TE) per g sample.

Oxygen radical absorbance capacity (ORAC) was determined as described by Floegel et al., 2011 with some modifications. Briefly, 25 µL diluted extract were mixed with 150 µL of 10 nM fluorescein into the microplate well and incubated for 30 min at 37 °C temperature. Twenty five microlitres AAPH (2,2-azobis (2-amidinopropane) dihydrochloride) (CAS: 2997-92-4, Cayman Chemical, Ann Arbor, MI, USA) solution was added to the pre-incubated reaction mixture. Fluorescence was measured (excitation 485 nm; emission 510 nm) from the bottom microplate every 60 s for a total of 60 min. Data analysed by using Omega MARS data analysis software (program version 3.02 R2, BMG Labtech, Mornington, Australia), in order to calculate antioxidant capacity, trolox was used as a standard and results were expressed as a mmol trolox equivalent (TE) per g sample.

### 2.8 InVitro Carbohydrate Digestion (Glycaemic Glucose Equivalent-GGE) Analysis

The in vitro digestion process was carried out with the method developed by Foschia, Peressini, Sensidoni, Brennan and Brennan, 2015 [28] and used by Gao, J.R. et al., 2016 [29]. The method estimates the glucose released from the cookie samples during enzymatic hydrolysis over 120 min to predict glycaemic response. In brief: digestions were held in 60 mL plastic pots placed on a controlled temperature stirring hot plate (IKA RT 15, IKA Werke GmbH & Co. KG, Mendelheim, Germany). The samples (0.5 g) were mixed with 30 mL of reverse osmosis water and kept at 37 °C for 10 min with constant stirring on a magnetic starrier. Pepsin solution (1 mL of 1 g pepsin in 10 mL 0.05 M hydrogen chloride (HCl) was added and incubated for 30 min at 37 °C. Aliquots (1 mL) were collected (time 0) from the digestion mixture and added to 4 mL alcohol to arrest enzyme reaction. Amyloglucosidase (0.1 mL) was added to the digestion mixture to prevent end product inhibition of pancreatic α-amylase. Then pancreatin solution (5 mL of 2.5% pancreatin in 0.1 M Malate buffer pH 6.0) was added to the mixture. Further 1 mL aliquots were collected at 20, 60 and 120 min and treated as before, then stored at 4 °C until reducing sugar analysis was carried out. The 3,5-dinitrosalicylic acid (DNS) method was followed to measure reducing sugar content of the samples during in vitro digestion. Glucose release was calculated in mg glucose/g sample and plotted against time and area under the curve (AUC) was calculated by dividing the graph into trapezoids.

### 2.9. Statistical Analysis

All data was analysed by using the data analysis software, Minitab (version 17, Minitab Inc., State College, PA, USA) to establish significant differences. Analysis of Variance (One-way) was employed with Tukey’s test at 95% confidence interval (*p* < 0.05) in all cases. All values were presented as the mean of triplicate determinations ± standard deviation.

## 3. Results and Discussion

Cookies were prepared using astaxanthin powder and wholemeal flour. The effects of astaxanthin powder replacement on the physical properties and functional properties of wholemeal flour cookies were analysed.

### 3.1. Physical Properties of Cookies

The physical characteristics of the cookies are summarised in Table 2. The results showed a significant reduction (*p* < 0.05) in the height and diameter gain of the cookies containing astaxanthin and a significant reduction of (*p* < 0.05) weight loss of the wheat and oat flour cookies with 15% addition of astaxanthin. As the amount of astaxanthin powder increased the weight loss, height and diameter decreased. The largest height changes were observed in cookies made from wholemeal wheat flour, and the largest diameter changes were observed in cookies made from wholemeal oat flour. This observation could be attributed to the hydrophilic nature of the ingredients [30]. The spread factor of a cookie is affected by dough viscosity as well as the acid-base reaction of the ingredients (sodium bicarbonate and fat), causing bubbles in the dough to expand in volume [31]. Physical evaluation of the cookies reported by [32,33], suggested that the spread factor is affected by the water holding capacity of the ingredients. Cookies made with wholemeal barley had increased moisture content with increasing astaxanthin addition. The reason for this phenomenon is that the physical state of starch, protein and fibre are the key determinants of the water holding capacity of the flour as suggested in other papers [34,35,36]. The moisture content of wheat, oat and barley cookies increased significantly (*p* < 0.05) at all levels of astaxanthin addition (Table 2). This can be attributed to differences in water holding capacity of the ingredients especially different flours [37]. Correspondingly, the hardness of the cookies decreased with the addition of astaxanthin (Table 2). The study indicated that when astaxanthin was incorporated into wheat and oat cookies they were softer and barley cookies were harder in comparison to control cookies. This suggests that water holding capacity of astaxanthin is intermediary between oat and barley flour and it could be due to the nature of the starch and starch-protein interface of different flour. The [38] found that differences in swelling behaviour of the starch granules resulted in cookies with different textural properties**,** while [34] showed that increased protein content affected the interaction of starch and protein and their hydrogen bonding during dough development.

### 3.2. Colour

The colour profile of the cookie samples (surface and ground) are summarised in Table 3. Both the surface colour and the total colour (represented by the ground sample) were measured to determine if there was any interaction in terms of food addition and colour enhancement. The addition of astaxanthin to three types of flour cookies significantly (*p* < 0.05) decreased the lightness (L*), causing the cookies to became red (a*) and decreased yellowness (b*). There was a significant colour change as illustrated by the △E value of the three kind of flour cookies in the following order: control >5% astaxanthin >10% astaxanthin >15% astaxanthin cookies. The main factor causing the colour change of the cookies is due to the pigment of astaxanthin powder, as the level of substitution increased lightness of the cookies decreased and greenness increased. However, the reaction between reducing sugars and amino acids (maillard reaction; starch dextrinization and caramelization) which is induced by heating during baking time also enhances darkness the cookie colour [39] as reflected in colour change (Table 3; △E value).

### 3.3. Total Phenolic Content (TPC) and Antioxidant Activity of Cookies

The phenolic content, DPPH radical scavenging and ORAC activity of the cookies are summarised in Table 4. It can be seen that the phenolic content increased significantly (*p* < 0.05), and proportionately, with the replacement of astaxanthin powder. This phenomenon is likely to be due to the high amount of phenolic compounds present in astaxanthin (10,000–40,000 mg/kg). Spiller and Dewell (2003) [40] and Sharma and Gujral (2014) [41] have shown that wheat flour has less phenolic compounds compared to barley and oat flour. ORAC values were observed to increase as astaxanthin increased in the formulation (Table 4). Increasing the level of astaxanthin in cookies resulted in a significant increase (*p* < 0.05) of DPPH scavenging activity. These results are due to the addition of astaxanthin derived from microalgae. Previous research has illustrated that astaxanthin compounds are 10 times stronger than the other carotenoids [20] in terms of phenolic antioxidant activities.

### 3.4. Glycaemic Glucose Equivalent (GGE) Analysis

**Figure 1:**
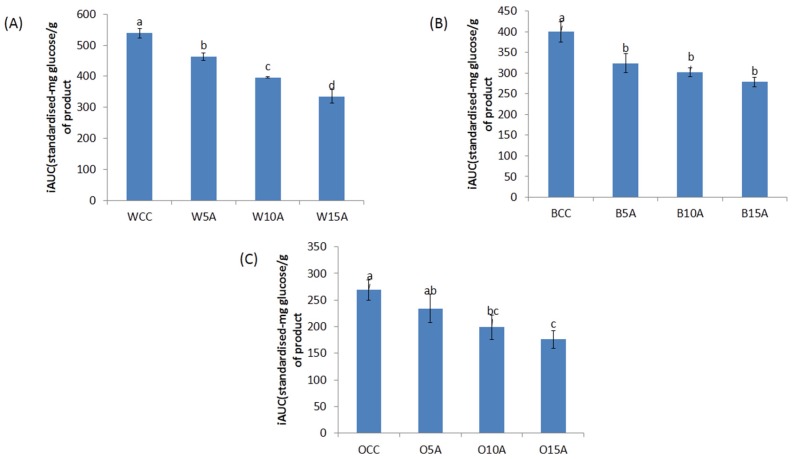
Reducing sugar released (mg/g sample) after 120 min digestion of (**A**) wheat, (**B**) barley and (**C**) oat wholemeal flour cookies with astaxanthin substitution. WCC, wheat cookie control; W5A, wheat + 5% astaxanthin cookie; W10A, wheat + 10% astaxanthin cookie; W15A, wheat + 5% astaxanthin cookie; BCC, barley cookie control; B5A, barley + 5% astaxanthin cookie; B10A, barley + 10% astaxanthin cookie; B15A, barley + 15% astaxanthin cookie; OCC, oat cookie control; O5A, oat + 5% astaxanthin cookie; O10A, oat + 10% astaxanthin cookie; O15A, oat + 15% astaxanthin cookie. (a–d), Means within same figure that do not share the same superscript are significantly different (*p* < 0.05).

Figure 1 illustrates the in vitro digestion of cookies, calculated as the amount of reducing sugar released by digestive enzymes over 120 min. All the samples demonstrated the impact of the substitution of astaxanthin in the following order (5% > 10% > 15%) and significantly slowed the amount of reducing sugar released (calculated as mg glucose/g sample of incremental area under the curve (iAUC)) as compare with the control cookies.

It is possible that the high antioxidant activity of the astaxanthin powder could be related to the decreased rate of sugar released [21]. Researchers have shown that antioxidants can impair enzyme activity during the digestion [42]. The interaction between phenolic compounds and digestive enzymes [43] could affect the non-covalent starch-phenolic interactions thus impeding starch degradation [44,45]. Additionally, the rate of sugar release may also be decreased due to the non-starchy network of fibre and protein in the system which entraps starch granules and acts as a physical barrier thus limiting enzyme accessibility [28,46].

**Figure 2:**
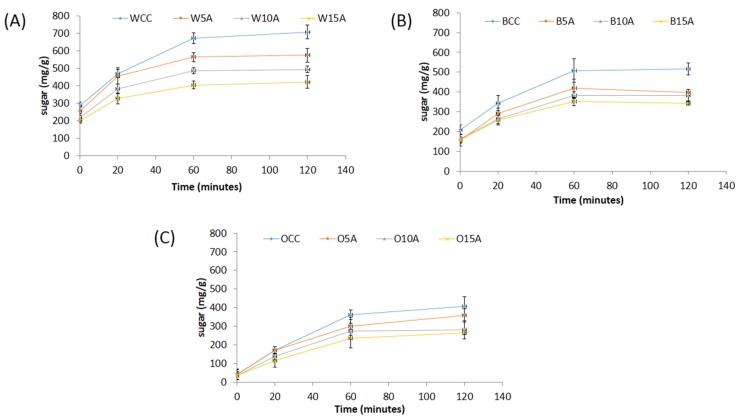
Reducing sugar released (mg/g sample) during the 120 min in vitro digestion process of (**A**) wheat, (**B**) barley and (**C**) oat wholemeal flour cookies with astaxanthin substitution. WCC, wheat cookie control; W5A, wheat + 5% astaxanthin cookie; W10A, wheat + 10% astaxanthin cookie; W15A, wheat + 5% astaxanthin cookie; BCC, barley cookie control; B5A, barley + 5% astaxanthin cookie; B10A, barley + 10% astaxanthin cookie; B15A, barley + 15% astaxanthin cookie; OCC, oat cookie control; O5A, oat + 5% astaxanthin cookie; O10A, oat + 10% astaxanthin cookie; O15A, oat + 15% astaxanthin cookie.

Figure 2 illustrates the rate of reaction of starch conversion to reducing sugar release over the 120 min in vitro digestion period. Form this figure it can be observed that the rate of reaction between 20–120 min appears to be greater for the control samples as compared with the samples containing astaxanthin. It can also be observed that the oat samples generally showed a lower sugar release profile than the barley and the wheat samples. It is possible that the in vitro digestion studies observed in Figure 1 and Figure 2 are related to the total phenolic content/antioxidant activity of the samples (Table 4). Further work is required to determine whether this is an indirect relationship or if there is a mechanistic association between phenolic content of the cookies and reduced starch digestion.

## 4. Conclusions

The research has illustrated the possible use of novel natural ingredients in alerting the functional quality and biological activity of simple foods. In particular, in vitro digestion (GGE analysis) of the cookies demonstrated significantly lower glucose release when astaxanthin increased in the formulation. The results also demonstrated that the combination of astaxanthin with wholemeal flour significantly improve the antioxidant properties of the cookies. Thus, the inclusion of astaxanthin illustrates a potential synergy between microalgae and wholemeal flour of the model food. As such this combination can contribute to the intake of natural bioactive compounds in the human diets for the potential health benefits.

## Figures and Tables

**Table 1:** Model food formulation.

Sample	Wholemeal Flour (g)	Astaxanthin Powder (g)	Other Ingredients
Control	225.00	-	Vegetable shortening (64.0 g), sugar (130 g), salt (2.1 g), sodium bicarbonate (2.5 g), dextrose solution (33 g), water (16 g)
5% Astaxanthin powder	213.75	11.25	Vegetable shortening (64.0 g), sugar (130 g), salt (2.1 g), sodium bicarbonate (2.5 g), dextrose solution (33 g), water (16 g)
10% Astaxanthin powder	202.50	22.50	
15% Astaxanthin powder	191.25	33.75	

**Table 2:** Physical characteristics (after baking: changes in height (%), diameter (%) and weight loss (%); moisture content (%) and hardness (kg) of the model cookies).

Sample	Increase in Height (%)	Increase in Diameter (%)	Weight Loss (%)	Moisture Content (%)	Hardness (kg)
WCC	94.39 ± 3.06 ^a^	3.93 ± 0.226	9.71 ± 0.04 ^a^	7.50 ± 0.11 ^c^	9.26 ± 0.13 ^a^
W5A	71.44 ± 8.39 ^b^	2.96 ± 1.139 ^b^	9.63 ± 0.02 ^a,b^	7.83 ± 0.01 ^b^	7.79 ± 0.16 ^b^
W10A	59.39 ± 3.06 ^b,c^	2.27 ± 0.216 ^b^	9.48 ± 0.12 ^a,b^	7.91 ± 0.07 ^b^	7.35 ± 0.58 ^b^
W15A	52.94 ± 0.75 ^c^	1.15 ± 0.925	9.44 ± 0.12 ^b^	8.21 ± 0.03 ^a^	7.06 ± 0.48 ^b^
BCC	94.33 ± 6.78 ^a^	5.23 ± 1.168	10.31 ± 0.11 ^a^	7.74 ± 0.02 ^d^	4.12 ± 0.12 ^c^
B5A	83.50 ± 1.04 ^a,b^	4.76 ± 0.444	10.41 ± 0.11 ^a^	7.79 ± 0.02 ^c^	4.98 ± 0.20 ^b^
B10A	74.61 ± 2.91 ^b,c^	3.67 ± 0.731 ^b^	10.46 ± 0.20 ^a^	7.90 ± 0.01 ^b^	5.26 ± 0.22 ^b^
B15A	65.50 ± 4.84 ^c^	2.72 ± 0.314	10.67 ± 0.28 ^a^	8.17 ± 0.02 ^a^	6.21 ± 0.10 ^a^
OCC	70.94 ± 0.91 ^a^	23.20 ± 0.25 ^a^	11.54 ± 0.17 ^a^	5.55 ± 0.05 ^d^	7.57 ± 0.05 ^a^
O5A	67.94 ± 2.46 ^a,b^	12.81 ± 0.49 ^a^	11.14 ± 0.23 ^a,b^	6.09 ± 0.04 ^c^	7.23 ± 0.14 ^a,b^
O10A	64.55 ± 0.25 ^b^	7.80 ± 0.05 ^c^	10.63 ± 0.22 ^b,c^	6.66 ± 0.04 ^b^	7.02 ± 0.12 ^b^
O15A	55.55 ± 0.91 ^c^	3.62 ± 0.14 ^d^	10.23 ± 0.23 ^c^	7.12 ± 0.09 ^a^	6.16 ± 0.23 ^c^

Data are presented as mean ± standard deviation, *n* = 3; (a–d): Means within same columns for same flour cookie group that do not share the same superscript are significantly different (*p* < 0.05). W, Wheat; B, Barley; O, Oat; CC, Cookie Control; A, Astaxanthin (5%, 10% or 15%).

**Table 3:** The CIE colour profiles of the cookies.

Sample	L*	a*	b*	△E
*Surface Cookie colour*				
WCC	90.40 ± 0.42 ^a^	−5.79 ± 0.35 ^a^	33.05 ± 0.09 ^a^	96.43 ± 0.44 ^a^
W5A	84.14 ± 0.26 ^b^	−7.37 ± 0.08 ^b^	29.16 ± 0.23 ^b^	89.36 ± 0.32 ^b^
W10A	82.20 ± 0.10 ^c^	−8.72 ± 0.20 ^c^	27.42 ± 0.06 ^c^	87.09 ± 0.13 ^c^
W15A	81.42 ± 0.32 ^c^	−8.27 ± 0.08 ^c^	26.45 ± 0.34 ^d^	86.01 ± 0.40 ^d^
BCC	94.43 ± 0.45 ^a^	−8.16 ± 0.76 ^a^	34.55 ± 0.27 ^a^	100.89 ± 0.58 ^a^
B5A	86.97 ± 0.19 ^b^	−9.25 ± 0.04 ^b^	32.16 ± 0.07 ^b^	93.19 ± 0.20 ^b^
B10A	84.77 ± 0.23 ^c^	−7.88 ± 0.25 ^a^	30.06 ± 0.15 ^c^	90.29 ± 0.27 ^c^
B15A	83.10 ± 0.14 ^d^	−7.83 ± 0.01 ^a^	27.95 ± 0.14 ^d^	88.02 ± 0.18 ^d^
OCC	91.31 ± 0.69 ^a^	−5.64 ± 0.34 ^a^	35.24 ± 0.15 ^a^	98.04 ± 0.71 ^a^
O5A	84.19 ± 0.14 ^b^	−7.63 ± 0.34 ^b^	30.64 ± 0.15 ^b^	89.91 ± 0.20 ^b^
O10A	82.21 ± 0.13 ^c^	−7.87 ± 0.13 ^b^	28.26 ± 0.11 ^c^	87.31 ± 0.17 ^c^
O15A	80.77 ± 0.15 ^d^	−8.17 ± 0.13 ^b^	26.56 ± 0.19 ^d^	85.39 ± 0.22 ^d^
*Ground Cookie colour*				
WCC	87.20 ± 0.20 ^a^	−0.32 ± 0.03 ^a^	45.33 ± 0.10 ^a^	97.55 ± 0.30 ^a^
W5A	77.82 ± 0.06 ^b^	−6.32 ± 0.04 ^c^	43.72 ± 0.26 ^b^	90.29 ± 0.01 ^b^
W10A	75.42 ± 0.22 ^c^	−6.98 ± 0.03 ^d^	38.31 ± 0.15 ^c^	84.88 ± 0.13 ^c^
W15A	69.10 ± 0.30 ^d^	−6.20 ± 0.02 ^b^	34.13 ± 0.12 ^d^	77.32 ± 0.22 ^d^
BCC	95.13 ± 0.07 ^a^	−13.41 ± 0.21 ^c^	43.14 ± 0.41 ^a^	105.32 ± 0.13 ^a^
B5A	82.96 ± 0.62 ^b^	−5.86 ± 0.23 ^a^	43.95 ± 0.65 ^a^	94.06 ± 0.30 ^b^
B10A	74.46 ± 0.63 ^c^	−6.56 ± 0.12 ^b^	44.02 ± 0.09 ^a^	86.74 ± 0.56 ^c^
B15A	71.13 ± 0.77 ^d^	−6.04 ± 0.38 ^a,b^	39.85 ± 1.38 ^b^	81.76 ± 0.24 ^d^
OCC	93.15 ± 0.59 ^a^	−9.32 ± 0.1.34 ^b^	49.41 ± 1.35 ^a^	105.86 ± 0.02 ^a^
O5A	85.92 ± 0.27 ^b^	−8.36 ± 0.09 ^a,b^	39.58 ± 0.21 ^b,c^	94.97 ± 0.16 ^b^
O10A	73.24 ± 0.36 ^c^	−7.33 ± 0.03 ^a^	36.04 ± 0.07 ^c^	81.96 ± 0.29 ^c^
O15A	71.19 ± 0.47 ^d^	−6.99 ± 0.57 ^a^	41.34 ± 2.68 ^b^	82.64 ± 0.97 ^c^

L*, lightness (0 = black, 100 = white); a*, red (+) to green (-); b*, yellow (+) to blue (-); △E, colour difference. Data are presented as mean ± standard deviation, *n* = 3; (a–d), Means within same columns for same flour cookie group that do not share the same superscript are significantly different (*p* < 0.05). W, Wheat; B, Barley; O, Oat; CC, Cookie Control; A, Astaxanthin (5%, 10% or 15%).

**Table 4:** Total phenolic content and antioxidant capacity.

Sample	TPC (mg GAE/g Sample)	DPPH (μmol TE/g Sample)	ORAC (mmol TE/g Sample)
WCC	0.59 ± 0.01 ^d^	0.54 ± 0.01 ^d^	0.09 ± 0.001 ^b^
W5A	0.80 ± 0.01 ^c^	0.95 ± 0.03 ^c^	0.11 ± 0.001 ^a^
W10A	0.95 ± 0.01 ^b^	1.10 ± 0.01 ^b^	0.12 ± 0.001 ^a^
W15A	1.14 ± 0.01 ^a^	1.26 ± 0.03 ^a^	0.12 ± 0.004 ^a^
BCC	0.63 ± 0.01 ^c^	1.36 ± 0.01 ^d^	0.08 ± 0.003 ^b^
B5A	0.95 ± 0.02 ^b^	1.69 ± 0.02 ^c^	0.09 ± 0.002 ^a^
B10A	1.15 ± 0.09 ^a^	1.74 ± 0.01 ^b^	0.09 ± 0.002 ^a^
B15A	1.27 ± 0.01 ^a^	1.79 ± 0.01 ^a^	0.10 ± 0.002 ^a^
OCC	0.87 ± 0.01 ^d^	1.13 ± 0.01 ^d^	0.08 ± 0.001 ^c^
O5A	1.03 ± 0.01 ^c^	1.22 ± 0.01 ^c^	0.10 ± 0.002 ^b^
O10A	1.28 ± 0.01 ^b^	1.34 ± 0.01 ^b^	0.10 ± 0.001 ^a^
O15A	1.44 ± 0.01 ^a^	1.46 ± 0.01 ^a^	0.11 ± 0.001 ^a^

Data are presented as mean ± standard deviation, *n* = 3; (a–d), Means within same columns for same flour cookie group do not share the same superscript are significantly different (*p* < 0.05). W, Wheat, B, Barley; O, Oat; CC, Cookie Control; A, Astaxanthin (5%, 10% or 15%).
